# fNIRS-Based characterization of adolescent depression using dynamic functional connectivity biomarkers in a verbal fluency task

**DOI:** 10.1186/s12888-026-07799-3

**Published:** 2026-01-17

**Authors:** Tongxin Li, Yeqing Dong, Qingyan Jiao, Lijun Chu, Jianli Qiu, Yanlei Fu, Yongyan Gao, Zhonghui Ma, Xia Sun, Yong Zhang

**Affiliations:** 1 Institute of Mental Health, Tianjin Anding Hospital, Tianjin, China; 2 Unit of Bipolar Disorder, Tianjin Anding Hospital, Tianjin, China; 3 Functional Department, Tianjin Anding Hospital, Tianjin, China; 4 Unit of Psychiatry Disorder, Tianjin Anding Hospital, Tianjin, China; 5 Department of Laboratory Medicine, Tianjin Anding Hospital, Tianjin, China

**Keywords:** Adolescents, Major depressive disorder, Functional near-infrared spectroscopy, Verbal fluency task, Dynamic functional connectivity, Principal components, Random forest

## Abstract

**Background:**

The human brain is a dynamic neural system with time-varying functional connectivity (FC) strengths between brain regions. Evidence indicates that adolescents with major depressive disorder (MDD) exhibit decreased average FC strength in cognitive tasks. Nevertheless, research focused on dynamic FC analysis in this population remains limited. This study aims to identify cognitive task-related dynamic FC features as valuable biomarkers to characterize clinical symptoms in adolescents with MDD.

**Methods:**

A total of 83 adolescents with MDD and 78 age/sex-matched healthy controls (HCs) were recruited. We utilized functional near-infrared spectroscopy (fNIRS) to record brain functional data from participants while they performed the verbal fluency task (VFT). An analytical framework for fNIRS data was proposed, in which the average FC strength values over the entire VFT duration and the principal components (PCs) of dynamic (time-varying) FC strength values were extracted as static and dynamic FC features, respectively. A random forest model was built to distinguish adolescents with MDD from HCs. Statistical analyses of the FC features were conducted to identify between-group differences, as well as their relationships with clinical symptoms in adolescents with MDD.

**Results:**

The random forest model achieved an accuracy of 86.32% (95% confidence interval: 83.75%-89.38%) for distinguishing adolescents with MDD from HCs. Significant between-group differences emerged in several FC features (false discovery rate-corrected *q* < 0.05). For adolescents with MDD, the average FC strength value in the right dorsolateral prefrontal cortex (DLPFC) ~ right medial prefrontal cortex (mPFC) pathway was a significant predictor of depressive and anxious symptoms; the 3rd PC of dynamic FC strength values in the left DLPFC ~ left temporal lobe (TL) pathway and the 5th PC of dynamic FC strength values in the right mPFC ~ right TL pathway were significant predictors of anhedonic symptoms.

**Conclusions:**

VFT-related static and dynamic FC features in specific brain pathways are potential biomarkers for characterizing clinical symptoms in adolescents with MDD. The developed random forest model holds promise as a diagnostic tool for MDD in adolescents.

**Clinical trial number:**

Not applicable.

**Supplementary Information:**

The online version contains supplementary material available at 10.1186/s12888-026-07799-3.

## Introduction

Major depressive disorder (MDD) represents a leading cause of psychiatric disability worldwide and is highly comorbid with anxiety disorders [1–3]. Adolescence is a period involving a high prevalence of MDD, with nearly 20% of adolescents suffering from MDD between the ages of 12 and 18 [4]. Cognitive impairments are core symptoms of MDD [5]; they are not merely behavioral symptoms of MDD but direct reflections of its underlying pathophysiology [6]. In the past 2 decades, neuroimaging findings have identified abnormal functional connectivity (FC) and activation patterns in cognitive tasks in adults (aged > 18 years) with MDD [7, 8]. Nevertheless, the adolescent brain is undergoing neural development [9], which means that neuroimaging findings on FC or activation from adults with MDD cannot be translated to adolescents with MDD. It is therefore essential to develop adolescent-specific neuroimaging biomarkers for characterizing clinical symptoms in this population.

In recent years, functional near-infrared spectroscopy (fNIRS) has emerged as a valuable tool to quantify brain activity. As an optical technique, fNIRS offers non-invasive and rapid measurements of hemoglobin concentration variations, making it particularly suitable for tracking brain functional changes during cognitive tasks [10–12]. In fNIRS studies, verbal fluency task (VFT) is the most commonly investigated cognitive task, because the VFT performance is affected by executive functions, verbal memory and psychomotor speed and is associated with activation in prefrontal and temporal regions [13]. Yet individuals with MDD typically exhibit hypoactivation in these two key brain regions [13]. However, the VFT-related brain activation metric (hemoglobin concentration variations) is not correlated with clinical symptoms in adolescents with MDD [14–16]. This prompts the need to examine VFT-related FC features as valuable biomarkers for this population.

Currently, the VFT-related FC features (or patterns) in adolescents with MDD are not fully investigated, as only one previous fNIRS study has reported a decrease in VFT-related average FC strength value within the prefrontal and temporal regions in this population [17]. It remains unknown whether the average FC strength values within these two brain regions in adolescents with MDD are correlated with their clinical symptoms. In addition, the derivation of the average FC strength value between brain regions typically employs seed correlation analysis [18]. The seed correlation analysis utilizes a single correlation coefficient to represent the average FC strength value between any two brain regions over the entire measurement period. Notably, VFT is a dynamic cognitive task that requires rapid, time-varying coordination of neural networks for successful execution [13]. Employing only the average FC strength values over the entire VFT period renders an oversimplified representation of FC features during cognitive tasks. Consequently, exploring dynamic FC analysis in MDD patients is crucial, as it may provide a more comprehensive understanding of the psychopathological mechanisms underlying cognitive impairments in MDD.

This study aimed to assess the utility of VFT-related dynamic FC features as biomarkers for characterizing clinical symptoms in adolescents with MDD. We proposed an analytical framework for fNIRS data that extracts the average FC strength values over the entire VFT period and the principal components (PCs) of dynamic (time-varying) FC strength values as static and dynamic FC features, respectively. A machine learning model was then built to distinguish adolescents with MDD from HCs. We also compared the FC features between healthy controls (HCs) and adolescents with MDD, and investigated the relationships between the FC features and clinical symptoms in adolescents with MDD. Notably, the current dynamic FC analysis commonly employs the unsupervised *k*-means clustering algorithm [19, 20], where the suitable cluster number is determined by the elbow criterion [21]. In principle, the elbow criterion remains fundamentally limited by its subjective interpretation of the inflection point [21]. In recent years, principal component analysis (PCA) has been widely used for extracting dynamic features (i.e., PCs) of time-varying series. PCA-derived PCs of electrophysiological or neuroimaging data have been applied to neurobiological interpretability, such as in characterizing the correlations between dopamine neuronal activity correlations and behavioral symptoms [22]. Nevertheless, the potential of PCA-derived PCs to provide neurobiological interpretability in the field of psychiatry remains insufficiently explored. In this study, we hypothesized that VFT-related PCs derived from dynamic FC strength values may correlate with clinical symptoms in adolescents with MDD, and aimed to provide the first evidence for their associations. We expect our work to be useful for the current diagnosis of MDD in adolescents beyond conventional psychiatric symptom-based assessments.

## Methods

### Participants

A total of 83 adolescents with MDD and 78 age/sex-matched HCs were recruited. The research protocol was approved by the Ethics Committee of Tianjin Anding Hospital, China (No. 2022-59). All participants and their guardians signed an informed consent form before participating in the study.

For adolescents with MDD, we recruited outpatients from Tianjin Anding Hospital based on the following inclusion criteria: 12–18 years old; diagnosis according to the DSM-5 criteria for MDD (at least two weeks of persistent depressed mood, loss of interest, or hopelessness co-occurred, with five additional symptoms affecting social working functioning); a minimum total score of 30 on the 21-item Depression Anxiety Stress Scale (DASS-21) and a minimum score of 14 on the depression subscale of the DASS-21 [23, 24]; Han Chinese. The exclusion criteria were as follows: any other psychoses on the DSM-5; organic brain diseases or other neurological diseases; treatment with physical therapies within the past 4 weeks; several physical diseases; a history of family abuse or bullying.

The HCs were recruited from Tianjin Anding Hospital based on the following inclusion criteria: 12–18 years old; no psychiatric symptoms or other mental disorders; Han Chinese; no physical diseases.

### Assessment of clinical symptoms

The study used the DASS-21 to assess clinical symptoms of the recruited adolescents with MDD [23]. The DASS-21 comprises three 7-item subscales measuring depression (DASS-D), anxiety (DASS-A), and stress (DASS-S) symptoms. The recruited adolescents with MDD were asked to rate each item’s applicability based on their past week’s experience on a 4-point scale ranging from 0 (did not apply to me at all) to 3 (applied to me very much or most of the time). For all the subscales, higher total scores indicate higher levels of depression, anxiety, and stress symptoms. Evidence has indicated cut-off thresholds of 30 on the DASS-21 total score and 10 on the DASS-D total score for the initial screening of MDD [24]. To enhance the diagnostic specificity for MDD screening, we implemented an optimized cut-off score of 14 on the DASS-D subscale.

We conducted an assessment of anhedonic symptoms using the Snaith-Hamilton Pleasure Scale (SHAPS) in a Chinese setting, because anhedonia is a significant component of depression that develops in adolescence [25]. The SHAPS is a 14-item questionnaire with self-statements of pleasure in response to hypothetical typically pleasant experiences that include sensory stimuli, social activities, and hobbies. Each item in the SHAPS is rated on 4 response categories ranging from 0 (strongly agree) to 3 (strongly disagree). A higher total SHAPS score indicates a higher level of anhedonic symptoms.

### Overall of the study

**Fig. 1:**
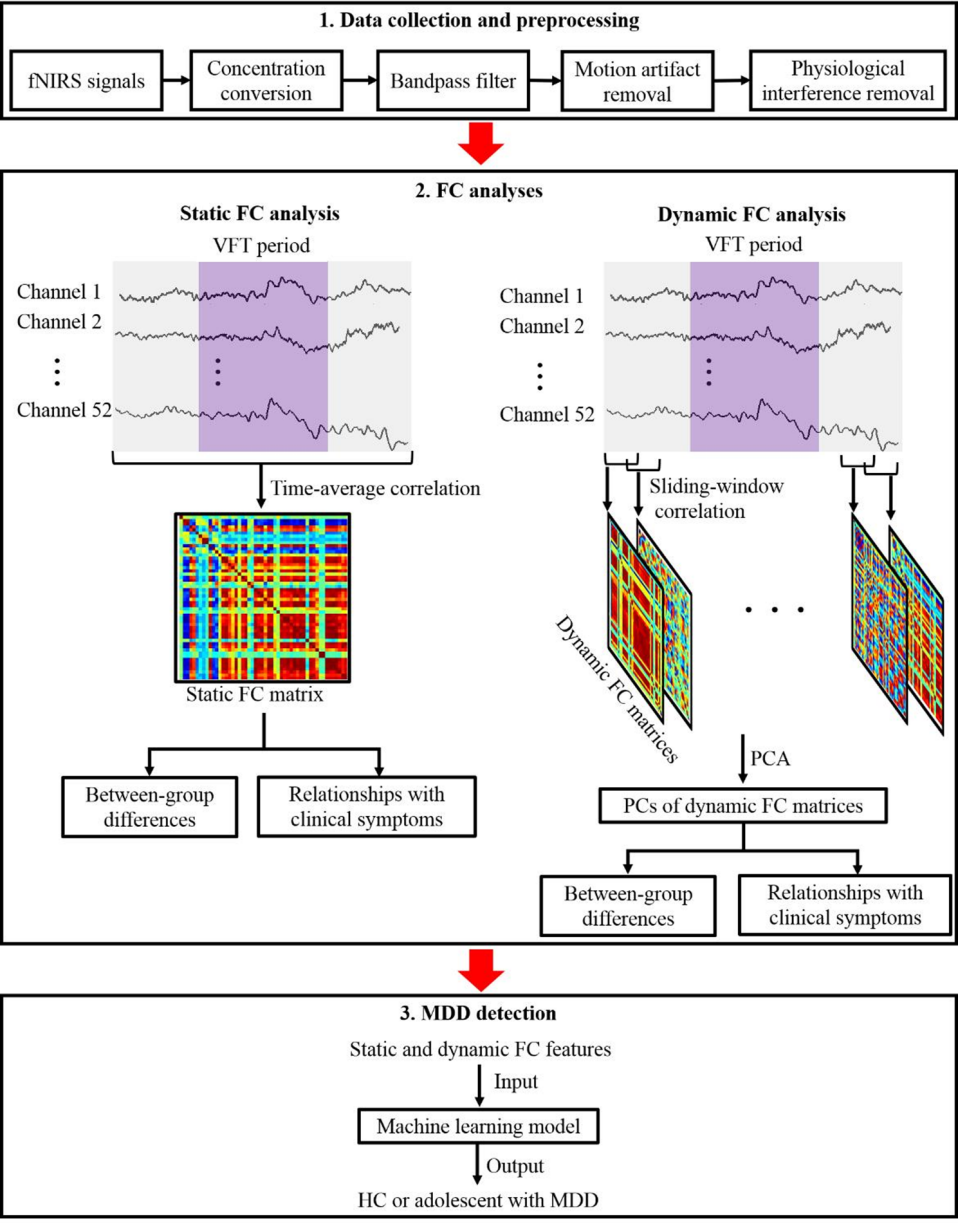
Flow of the study. Abbreviations: fNIRS, functional near-infrared spectroscopy; VFT, verbal fluency task; FC, functional connectivity; PCA, principal component analysis; PCs, principal components; MDD, major depressive disorder; HC, healthy control

The study flow comprises 3 main components (Fig. 1). (1) Data collection and preprocessing: a 52-channel fNIRS system was employed to collect fNIRS signals (absorption of near-infrared light) per participant during a VFT, and a preprocessing procedure was conducted to extract the oxyhemoglobin concentration information. (2) FC analyses: an analytical framework for fNIRS signals was presented. Time-averaging correlation analysis over the entire VFT period was employed to generate a static FC matrix per participant, representing average FC strength values between any two fNIRS channels. Sliding-window correlation analysis over the VFT period was employed to generate dynamic FC matrices per participant, representing dynamic FC strength values between any two fNIRS channels. PCA was then applied to extract PCs of the dynamic FC matrices. The extracted average FC strength values and PCs were considered as static and dynamic FC features, respectively. Statistical analyses were conducted on the extracted FC features to identify between-group differences, and to examine relationships between the FC features and clinical symptoms in adolescents with MDD. (3) MDD detection: a machine learning model was built to distinguish adolescents with MDD from HCs, using the extracted FC features.

### Data collection, quality control, and preprocessing

#### Data collection

**Fig. 2:**
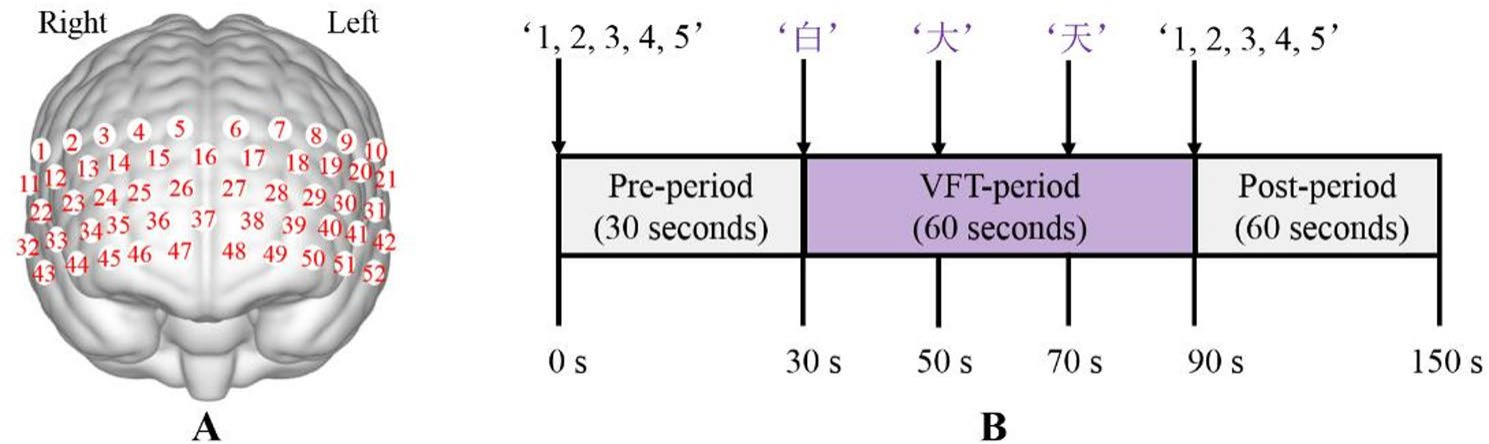
Illustration of brain functional data collection during VFT. **A** Schematic representation of the 52 fNIRS channels. The brain region map is presented from a frontal view. **B** Overview of the VFT. Abbreviations: fNIRS, functional near-infrared spectroscopy; VFT, verbal fluency task

A continuous-wave fNIRS system (ETG-4100, Hitachi) was used to measure hemoglobin concentration variations. The fNIRS system recorded the absorption of near-infrared light at dual wavelengths of 695 nm and 830 nm with a sampling rate of 10 Hz. According to the 10–20 electroencephalography system [26], we placed 33 probes (17 sources and 16 detectors) in an fNIRS cap to cover the prefrontal and temporal regions (between-probe distance of 3 cm), resulting in a total of 52 channels (Fig. 2A). The participants wore the fNIRS cap, sat in a chair, and performed a fixed VFT. The VFT comprised three periods: Pre-period, VFT-period, and Post-period (Fig. 2B). During the 30-second Pre-period, the participants were required to repeatedly continue counting from 1 to 5. Throughout the 60-second VFT period, the participants were instructed to construct multiple phrases using three Chinese characters: ‘白’, ‘大’, and ‘天’. In the 60-second Post-period, the participants were further required to repeatedly continue counting from 1 to 5. All participants were instructed to keep their heads still throughout the data collection process.

#### Data quality control

Data quality control was ensured through a combination of automated and manual procedures. Before each participant executed the VFT, the fNIRS system performed a self-check by incrementally increasing source power to optimize dynamic range and prevent detector saturation. Channels that failed the self-check were marked on the control panel, primarily due to the poor optical contact between probes and scalp. We then physically parted the hair beneath the probes to improve scalp contact. This manual procedure was repeated until all channels passed the self-check. Subsequently, 10-second fNIRS baseline signals per participant were recorded, and the signal-to-noise ratio (SNR) for each channel at the wavelength *λ* (= 695–830 nm) was determined by [27]

1 $${\mathrm{SNR}}_{{channel}}^{{{\lambda _{}}}}=20{\log _{10}}(\mu _{{channel}}^{{{\lambda _{}}}}/\sigma _{{channel}}^{{{\lambda _{}}}})$$

where $\mu _{{channel}}^{{{\lambda _{}}}}$ and $\sigma _{{channel}}^{{{\lambda _{}}}}$ denote the mean and standard deviation of the 10-second fNIRS baseline signals, respectively. Based on a previous report on investigating SNR variations with between-probe distance [28], we implemented a SNR threshold of 40 decibel to select high SNR channels. Evaluation of the 10-second fNIRS baseline signals across participants confirmed that channels 1, 10, 12, 20, 22, and 31 consistently exhibited SNRs below the 40 decibel threshold in at least one wavelength, probably due to the low detected light intensity resulting from their peripheral probe placement. These 6 channels were therefore excluded, retaining 46 channels for subsequent analyses.

#### Data preprocessing

The preprocessing of fNIRS signals acquired during the VFT was conducted using MATLAB software (2022a, MathWorks, USA) with four steps. (1) Concentration conversion: the modified Beer-Lambert law was used to transform the fNIRS signals into oxyhemoglobin concentration change (ΔHbO) and deoxyhemoglobin concentration change at the dual wavelengths. (2) Bandpass filter: a Butterworth bandpass filter with a frequency band of 0.01 to 0.2 Hz was employed to remove high frequencies (such as heartbeats) and baseline drift. (3) Motion artifact removal: the wavelet-based motion correction with the Daubechies 5 and a probability threshold of 0.1 was employed to remove artifacts due to body motion [29]. (4) Physiological interference removal: the hemodynamic modality separation method was utilized to eliminate physiological interference [30]. We selected ΔHbO signals as the backbone signals, because oxyhemoglobin information is associated with the presence of psychopathology and is more closely related to the blood oxygenation level measured by fNIRS [31].

According to the Brodmann brain localization system [32], the retained 46 fNIRS channels were divided into 6 regions of interest (ROIs), including right and left dorsolateral prefrontal cortices (DLPFC(R), DLPFC(L)), right and left temporal lobes (TL(R), TL(L)), and right and left medial prefrontal cortices (mPFC(R), mPFC(L)). The ΔHbO signal within each ROI was calculated through linear summation of the ΔHbO signals from the covered fNIRS channels. This ROI-based approach yielded a more robust and comparable feature set, which enables direct comparison with existing literature and improves neurophysiological interpretability [33].

### Extraction of FC features

#### Average FC strength values

For each individual data set, we employed the time-average correlation analysis over the entire VFT period. The normalized average FC strength value between any two ROIs was calculated using Pearson correlation analysis and Fisher’s z-transformation [34]. This procedure yielded a 6 × 6 static FC matrix with 15 (6 × 5 / 2) pairs of average FC strength values.

#### PCs of dynamic FC strength values

For each individual data set, we employed a 20-second sliding-time window and a 1-second increment along the VFT period. This dynamic parameter set was chosen to provide a good trade-off between false fluctuations and over-smoothing [19, 35]. The normalized FC strength values within each sliding-time window were calculated using Pearson correlation analysis and Fisher’s z-transformation [34]. Mathematically, the 150-second VFT period, 20-second sliding-time window, and 1-second increment yielded 131 dynamic FC matrices. Each matrix contained 15 pairs of dynamic FC strength values between any two ROIs. Subsequently, PCA was adopted to extract features of the 131 dynamic FC matrices.

PCA uses a vector space transformation to reduce the dimensionality of the data set into a smaller number of variables called PCs, which are linear combinations of the original variables [36]. We employed the PCA function in the MATLAB software. For each participant, the extracted 15 pairs of dynamic FC strength values were created into a *K* (= 15, the number of pairs of ROIs) × *M* (= 131, the number of sliding time windows) matrix **X**. To obtain PCs, we first calculated the covariance matrix of **X**, and then computed the eigenvalue *κ*_*m*_ (*m* = 1, 2, …, *M*) and eigenvector matrix **U** (= *u* ^(1)^, *u* ^(2)^, …, *u* ^(*M*)^) of the covariance matrix using singular value decomposition [37]. Here, *κ*_*m*_ determined the appropriate number of PCs, denoted as *N*, based on the criterion that the cumulative contribution rate (CCR) of the first *N* eigenvalues in *κ*_*m*_ reached 90–95%, which ensured that a significant portion of the total variance was retained while minimizing dimensionality [38]. According to PCA theory [38], the CCR was obtained via the following equation:

2 $$CCR=\sum\nolimits_{{n=1}}^{N} {({\kappa _n}} /\sum\nolimits_{{n=1}}^{M} {{\kappa _n}}){\kern 1pt} {\kern 1pt} \times {\text{100\% }},{\kern 1pt} {\kern 1pt} {\kern 1pt} 1 \leqslant N \leqslant M$$

To reduce the *M*-dimensional matrix **X** to *N*-dimensions, the first *N* columns of **U** were extracted to obtain the dimensionality reduction matrix **U**_reduce_ (= *u* ^(1)^, *u* ^(2)^, …, *u* ^(*N*)^), and the original matrix **X** was multiplied with **U**_reduce_ to obtain the final reduced matrix:

**D** = **XU**_reduce_ (3)

In the reduced matrix **D**, rows represented the order number of the 15 pairs of dynamic FC strength values, and columns represented the first *N* PCs of the dynamic FC strength values. Notably, the elements in **D** can be both positive and negative, which may complicate data interpretation and analysis. To address this issue, we concatenated all participants’ **D** matrices to generate a 2D matrix and then applied the shifting method by subtracting the minimum value in the 2D matrix [39]. This procedure ensured that the elements in **D** remained meaningful while preserving the original relationships.

### Statistical analyses

All statistical analyses were performed in SPSS software (20.0, IBM Corporation, USA), with a significance threshold at *p* < 0.05. Kolmogorov-Smirnov one-sample test was used to determine whether the statistical data were normally distributed. Normally distributed data were expressed as means ± standard deviations ($\bar {x}$± *s*), and *t*-test was used for comparison between groups. Non-normally distributed data were expressed as medians, and Wilcoxon rank-sum test was employed for comparison between groups. Count data were expressed as frequencies, and χ^2^ test was employed for comparison between groups. Multiple comparison correction was performed using the Benjamini-Hochberg false discovery rate (FDR) correction, with a significance threshold at *q* < 0.05.

Spearman correlation analyses were utilized to examine the relationships between the FC features and clinical symptom scores in adolescents with MDD. Multiple linear regression analyses were conducted to control for the potential confounding effects of age, gender, duration, and total antidepressant dosage on the clinical symptom scores. To standardize antidepressant dosage, fluoxetine equivalents were calculated for the 83 adolescents with MDD by the following equation [40]: fluoxetine = 40 mg/day; sertraline = 98.5 mg/day; paroxetine = 34 mg/day; clomipramine = 116.1 mg/day; venlafaxine = 149.4 mg/day. Prior to the multiple linear regression analyses, we conducted necessary diagnostics, including checks for linearity, normality, multicollinearity, and homoscedasticity.

### Distinguishing adolescents with MDD from HCs

The extracted static and dynamic FC features served as model input to distinguish adolescents with MDD from HCs. Random forest (RF) model was selected due to its effectiveness in dealing with limited sample sizes, addressing weak classification, and enhancing robustness against overfitting [41]. In our work, the RF model was configured to employ Gini impurity as the splitting criterion, and the out-of-bag bootstrap method was employed to determine the feature importance [42].

Model training, validation, and testing were performed using the MATLAB software. To maximize the use of the available dataset of the participants, we employed a 10-fold cross-validation method to divide the dataset into 10 subsets [43]. Within each fold, one distinct subset served as the testing set, one distinct subset served as the validation set, and the remaining eight subsets served as the training set. This division effectively reduced the impact of randomness and enabled a more accurate assessment of the model generalization to unseen data [43].

To determine the optimal hyper-parameter configuration and to reduce the risk of model underfitting or overfitting, a grid search was performed during the 10-fold cross-validation process [44]. The grid search space included the number of decision trees (10 to 150 in steps of 10), the minimum leaf size (1 to 11 in steps of 1), and the bootstrap fraction (0.5 to 1.0 in steps of 0.1). The maximum decision tree depth was excluded from the grid search as it was effectively controlled by the minimum leaf size. By applying the grid search, the optimal hyper-parameter configuration was selected based on the highest average classification accuracy on the validation sets across the 10-fold cross-validation process. With this optimal hyper-parameter configuration, the model performance within each fold was rigorously assessed on its corresponding testing set using 3 metrics: classification accuracy, F1-score, and the area under the curve of the receiver operating characteristic (AUC). The 95% confidence interval (CI) for these 3 metrics was calculated using the bootstrap method with 1000 iterations, as this number typically yielded stable and reliable estimates of CI and standard errors [45].

## Results

### General information of the participants

Table 1 presents the demographic and clinical data of the participants. There were no between-group differences in age, sex, or education (*p* > 0.05).

**Table 1:** Demographic and clinical data of the participants

Variables	Adolescents with MDD (*n* = 83)	HCs (*n* = 78)	t /χ2	*p*
Age (years)	15.48 ± 1.71	15.62 ± 1.77	-0.453	0.651
Sex (male / female)	37 / 46	36 / 42	0.040	0.841
Education (year)	8.52 ± 1.84	8.73 ± 1.60	-0.704	0.483
DASS-D score	16.12 ± 1.93	-	-	-
DASS-A score	11.43 ± 4.16	-	-	-
DASS-S score	11.22 ± 5.77	-	-	-
SHAPS score	33.07 ± 7.37	-	-	-

Abbreviations: MDD, major depressive disorder; HCs, healthy controls; DASS-D, depression items in the 21-item Depression Anxiety Stress Scale (DASS-21); DASS-A, anxiety items in the DASS-21; DASS-S, stress items in the DASS-21; SHAPS, Snaith-Hamilton Pleasure Scale

### Between-group differences in FC features

**Fig. 3:**
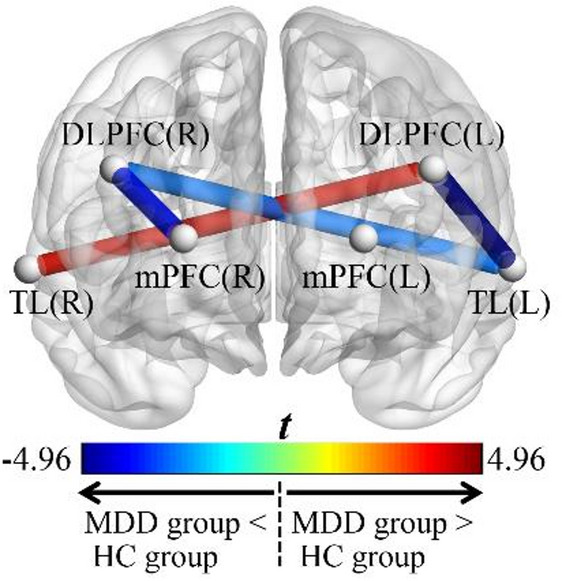
*T*-statistic map of between-group comparisons for average FC strength values, showing only significant results (FDR-corrected *q* < 0.05). Note: The brain region map is presented from a frontal view, colors reflect both the magnitude and directionality of the between-group differences (cooler colors: MDD group < HC group; warmer colors: MDD group > HC group). Abbreviations: MDD, major depressive disorder; HC, healthy control; FC, functional connectivity; FDR, false discovery rate; DLPFC, dorsolateral prefrontal cortex; mPFC, medial prefrontal cortex; TL, temporal lobe; R, right; L, left

Figure 3 shows the *t*-statistic map derived from between-group comparisons of the average FC strength values, with only significant results (FDR-corrected *q* < 0.05) displayed. The complete statistical results are presented in Supplemental Table 1. Compared with HCs, adolescents with MDD exhibited lower average FC strength values in the DLPFC(R) ~ mPFC(R), DLPFC(R) ~ TL(L), and DLPFC(L) ~ TL(L) pathways, and exhibited a higher average FC strength value in the DLPFC(L) ~ TL(R) pathway. The mean ROI-to-ROI average FC strength values were 0.32 for adolescents with MDD (SD = 0.11) and 0.39 for HCs (SD = 0.07), respectively, and the HCs had a higher mean ROI-to-ROI average FC strength value than that of adolescents with MDD (*t* = 2.82, *p* = 0.008).

**Fig. 4:**
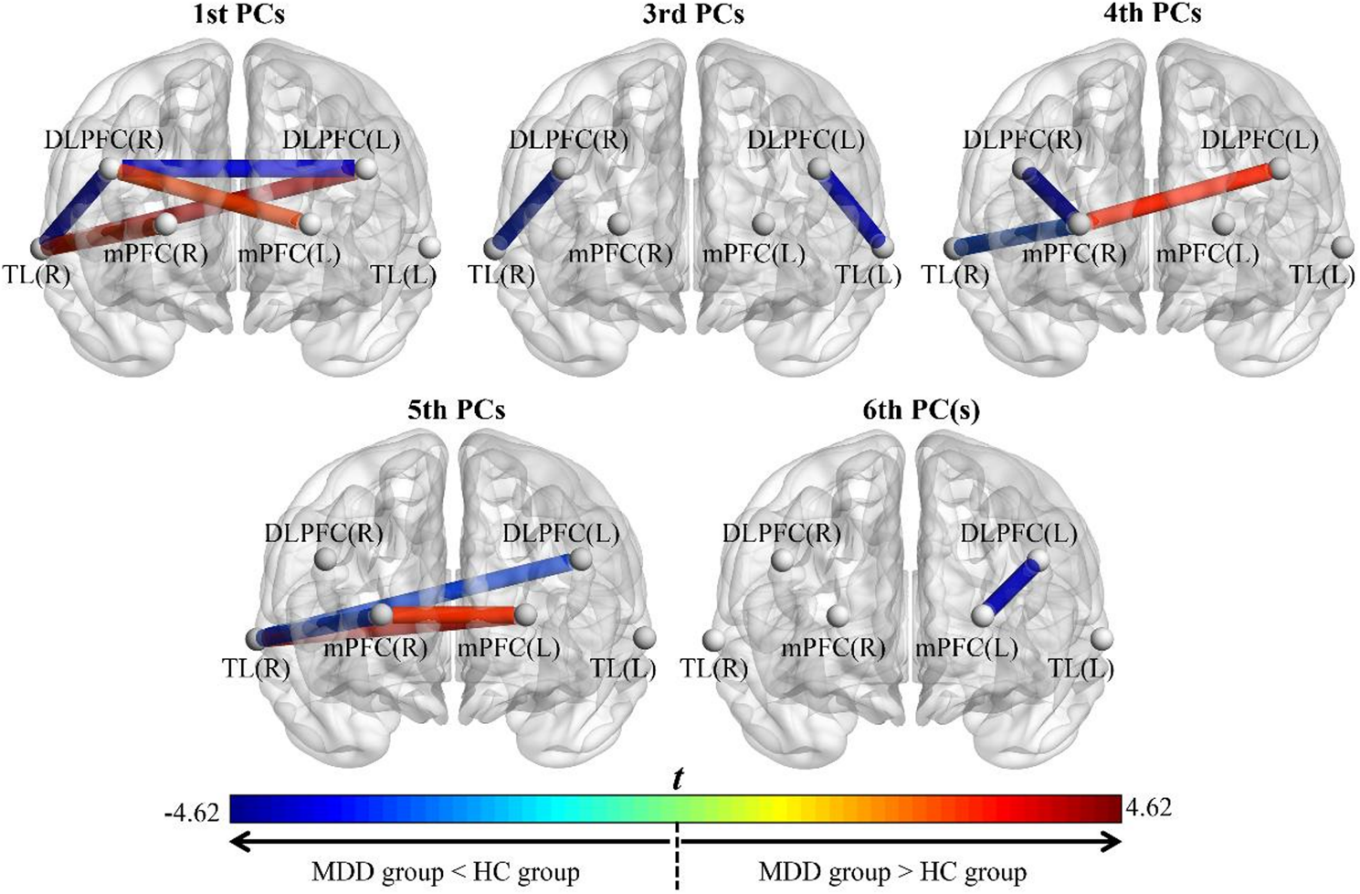
*T*-statistic maps of between-group comparisons for the first 6 PCs of dynamic FC strength values, showing only significant results (FDR-corrected *q* < 0.05). Note: The brain region map is presented from a frontal view, colors reflect both the magnitude and directionality of the between-group differences (cooler colors: MDD group < HC group; warmer colors: MDD group > HC group). Abbreviations: MDD, major depressive disorder; HC, healthy control; FC, functional connectivity; FDR, false discovery rate; DLPFC, dorsolateral prefrontal cortex; mPFC, medial prefrontal cortex; TL, temporal lobe; R, right; L, left

The appropriate number of PCs to present primary variables of dynamic FC matrices was 6, because the CCRs of the first 6 eigenvalues were determined to be 92.13 ± 3.33% for adolescents with MDD and 91.98 ± 2.03% for HCs, respectively. Figure 4 shows the *t*-statistic map derived from between-group comparisons of the first 6 PCs of dynamic FC strength values, with only significant results (FDR-corrected *q* < 0.05) displayed. The complete statistical results are presented in Supplemental Tables 2–7. Compared with HCs, adolescents with MDD exhibited these abnormalities in dynamic FC features (FDR-corrected *q* < 0.05): (1) lower 1st PCs of dynamic FC strength values in the DLPFC(R) ~ TL(R) and DLPFC(R) ~ DLPFC(L) pathways, and higher 1st PCs of dynamic FC strength values in the DLPFC(R) ~ mPFC(L), mPFC(R) ~ TL(R), and DLPFC(L) ~ TL(R) pathways; (2) lower 3rd PCs of dynamic FC strength values in the DLPFC(R) ~ TL(R) and DLPFC(L) ~ TL(L) pathways; (3) lower 4th PCs of dynamic FC strength values in the DLPFC(R) ~ mPFC(R) and mPFC(R) ~ TL(R) pathways, and a higher 4th PC of dynamic FC strength values in the DLPFC(L) ~ mPFC(R) pathway; (4) lower 5th PCs of dynamic FC strength values in the mPFC(R) ~ TL(R) and DLPFC(L) ~ TL(R) pathways, and higher 5th PCs of dynamic FC strength values in the mPFC(L) ~ TL(R) and mPFC(R) ~ mPFC(L) pathways; (5) a lower 6th PC of dynamic FC strength values in the DLPFC(L) ~ mPFC(L) pathway.

### Relationships between FC features and MDD clinical symptoms

DASS-D (all depression cases > clinical threshold of 14) and DASS-A (76 anxiety cases > clinical threshold of 7) were included in the correlation analyses, while DASS-S (19 stress cases > clinical threshold of 14) was excluded. The SHAPS score, although lacking predefined clinical thresholds unlike the DASS-21, was included in the correlation analyses because the recruited adolescents with MDD had significantly higher SHAPS scores (33.07 ± 7.37) than those of HCs (21.50 ± 5.17) reported in a previous study of college students [25].

**Fig. 5:**
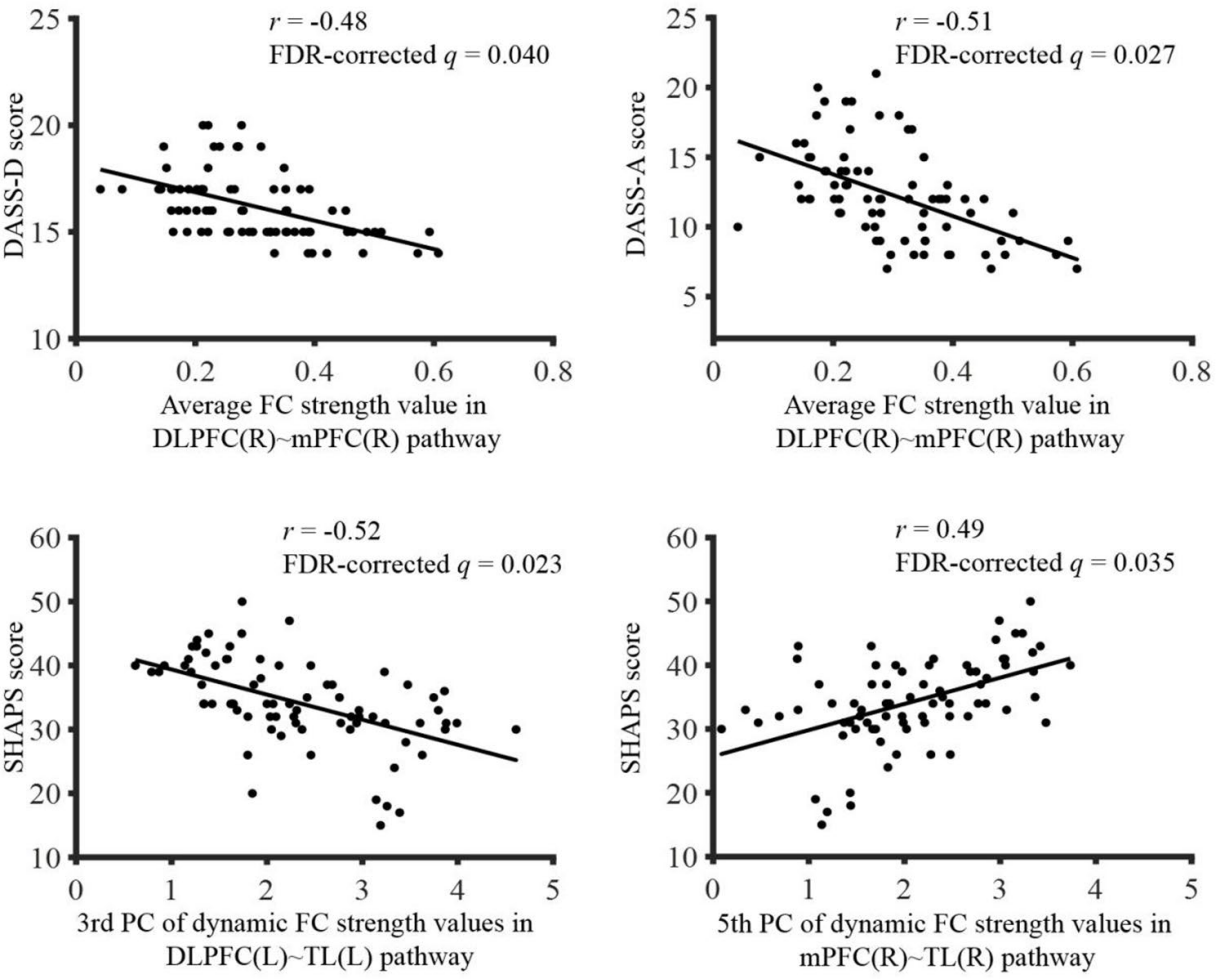
Significant correlations (FDR-corrected *q* < 0.05) between FC features and clinical symptom scores in adolescents with MDD. Note: Data were analyzed by Spearman correlation analyses. Abbreviations: DASS-D, depression items in the 21-item Depression Anxiety Stress Scale (DASS-21); DASS-A, anxiety items in the DASS-21; SHAPS, Snaith-Hamilton Pleasure Scale; FC, functional connectivity; FDR, false discovery rate; PC, principal component; DLPFC, dorsolateral prefrontal cortex; mPFC, medial prefrontal cortex; TL, temporal lobe; R, right; L, left

Spearman correlation analyses revealed these associations passed the Benjamini-Hochberg correction (FDR-corrected *q* < 0.05; Fig. 5): (1) the DASS-D/A scores and the average FC strength value in the DLPFC(R) ~ mPFC(R) pathway; (2) the SHAPS score and the 3rd PC of dynamic FC strength values in the DLPFC(L) ~ TL(L) pathway; (3) the SHAPS score and the 5th PC of dynamic FC strength values in the mPFC(R) ~ TL(R) pathway. The accurate correlation coefficients (*r*) and FDR-corrected *q* values are presented in Table 2.

**Table 2:** Calculated (*r*, FDR-corrected *q*) pairs for significant FC features and clinical symptom scores in adolescents with MDD

FC feature	Brain pathway	DASS-D score	DASS-A score	SHAPS score
Average FC strength values	DLPFC(R) ~ mPFC(R)	**(-0.48**,** 0.040)**	**(-0.51**,** 0.027)**	(-0.27, 0.431)
	DLPFC(R) ~ TL(L)	(-0.32, 0.759)	(-0.31, 0.112)	(-0.11, 0.595)
	DLPFC(L) ~ TL(R)	(0.20, 0.375)	(0.02, 0.922)	(0.09, 0.604)
	DLPFC(L ~ TL(L)	(0.04, 0.896)	(-0.27, 0.247)	(-0.25, 0.266)
PCs of dynamic FC strength values	1st in DLPFC(R) ~ TL(R)	(0.08, 0.671)	(-0.40, 0.066)	(-0.21, 0.331)
	1st in DLPFC(R) ~ DLPFC(L)	(-0.03, 0.907)	(-0.27, 0.247)	(-0.02, 0.922)
	1st in DLPFC(R) ~ mPFC(L)	(0.19, 0.363)	(0.14, 0.446)	(-0.02, 0.922)
	1st in mPFC(R) ~ TL(R)	(-0.14, 0.446)	(0.12, 0.543)	(0.11, 0.595)
	1st in DLPFC(L) ~ TL(R)	(-0.22, 0.303)	(0.20, 0.362)	(0.01, 0.995)
	3rd in DLPFC(R ~ TL(R)	(0.17, 0.384)	(0.02, 0.922)	(-0.08, 0.671)
	3rd in DLPFC(L) ~ TL(L)	(0.05, 0.759)	(-0.04, 0.856)	**(-0.52**,** 0.023)**
	4th in DLPFC(R) ~ mPFC(R)	(0.04,0.857)	(-0.17, 0.384)	(0.04, 0.879)
	4th in mPFC(R) ~ TL(R)	(0.03, 0.907)	(-0.02, 0.922)	(0.33, 0.099)
	4th in DLPFC(L) ~ mPFC(R)	(-0.31, 0.123)	(-0.31, 0.112)	(-0.22, 0.303)
	5th in mPFC(R) ~ TL(R)	(0.11, 0.595)	(-0.20, 0.362)	**(0.49**,** 0.035)**
	5th in DLPFC(L) ~ TL(R)	(-0.14, 0.446)	(0.06, 0.701)	(0.12, 0.545)
	5th in mPFC(L) ~ TL(R)	(-0.11, 0.555)	(-0.01, 0.995)	(-0.29, 0.227)
	5th in mPFC(R) ~ mPFC(L)	(-0.02, 0.922)	(-0.42, 0.059)	(0.20, 0.375)
	6th in DLPFC(L) ~ mPFC(L)	(-0.23, 0.298)	(-0.09, 0.604)	(-0.27, 0.247)

Data were analyzed by Spearman correlation analysis. Significant results (FDR-corrected *q <* 0.05) are highlighted in bold. Abbreviations: FDR, false discovery rate; FC, functional connectivity; PCs, principal components; DASS-D, depression items in the 21-item Depression Anxiety Stress Scale (DASS-21); DASS-A, anxiety items in the DASS-21; DASS-S, stress items in the DASS-21; SHAPS, Snaith-Hamilton Pleasure Scale; DLPFC, dorsolateral prefrontal cortex; mPFC, medial prefrontal cortex; TL, temporal lobe; R, right; L, left

Multiple linear regression analyses revealed that the regression models significantly predicted the DASS-D score (adjusted *R*^2^ = 0.303, *p* = 0.016), the DASS-A score (adjusted *R*^2^ = 0.271, *p* = 0.025), and the SHAPS score (adjusted *R*^2^ = 0.429, *p* = 0.003) in adolescents with MDD. Specifically, the average FC strength value in the DLPFC(R) ~ mPFC(R) pathway was a significant negative predictor of the DASS-D score (*β* = -0.502, *t* = -3.076, *p* = 0.005) and the DASS-A score (*β* = -0.545, *t* = -3.267, *p* = 0.003). Furthermore, the 3rd PC of dynamic FC strength values in the DLPFC(L) ~ TL(L) pathway was a significant negative predictor of the SHAPS score (*β* = -0.380, *t* = -2.316, *p* = 0.030), the 5th PC of dynamic FC strength values in the mPFC(R) ~ TL(R) pathway was a significant positive predictor of the SHAPS score (*β* = 0.454, *t* = 2.849, *p* = 0.009).

### Evaluation of RF model performance and feature importance

**Fig. 6:**
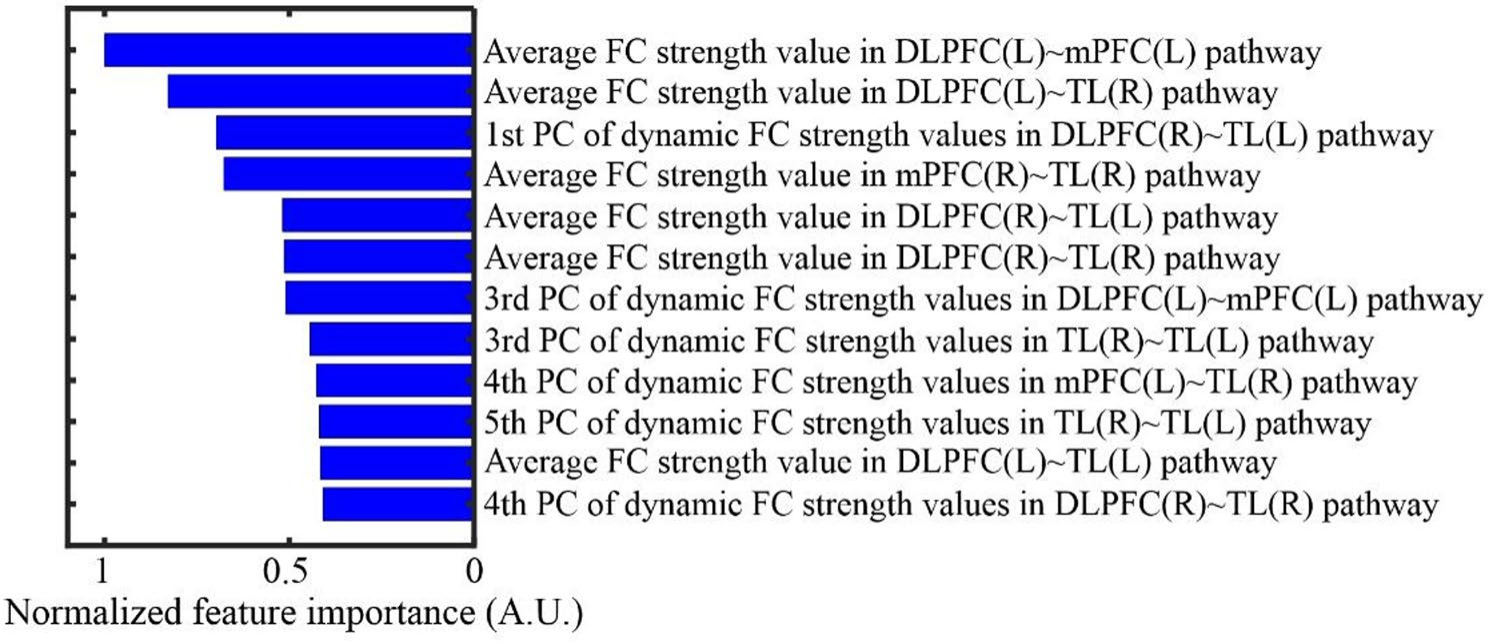
Top 12 normalized feature importance in the RF model with the optimal hyper-parameter configuration. Note: The feature importance for each fold of the cross-validation was calculated using the out-of-bag bootstrap method, and the resulting values were then averaged and normalized. Abbreviations: FC, functional connection; PC, principal component; DLPFC, dorsolateral prefrontal cortex; mPFC, medial prefrontal cortex; TL, temporal lobe; R, right; L, left

The grid search identified the optimal hyper-parameter configuration as follows: 70 decision trees, a minimum leaf size of 1, and a bootstrap fraction of 0.8. With this optimal hyper-parameter configuration, the RF model achieved a classification accuracy of 86.32% (95% CI: 83.75%-89.38%), an F1-score of 0.87 (95% CI: 0.83–0.90), and an AUC of 0.88 (95% CI: 0.86–0.90) on the testing sets across the 10-fold validation process. Figure 6 displays the top 12 normalized feature importance in the RF model, calculated using the out-of-bag bootstrap method [42]. A higher normalized feature importance means that the feature has a positive impact on enhancing the model performance [42]. As shown in Fig. 6, the top 12 features comprise 6 static and 6 dynamic FC features, indicating that both types of FC features contributed equally and primarily to the improvement of model performance.

## Discussions

This study proposed an analytical framework that jointly employed time-average correlation, sliding-window correlation, and PCA for extracting static and dynamic FC features from fNIRS data. It represented the first comprehensive investigation of the relationships between VFT-related FC features and clinical symptoms in adolescents with MDD. It was also the first application of the machine learning model using VFT-related FC features to distinguish adolescents with MDD from HCs. The statistical analyses indicated that adolescents with MDD exhibited several abnormal FC features, and certain FC features were significant predictors of their clinical symptom scores. The experimental findings demonstrated that the RF model was able to distinguish adolescents with MDD from HCs with a high accuracy of 86.32% (95% CI: 83.75%-89.38%).

Our study demonstrated a decrease in VFT-related average FC strength within the prefrontal and temporal regions in adolescents with MDD. This finding aligns with a previous VFT-based FC study investigating adolescent depression [17]. More specifically, our experimental results demonstrated that adolescents with MDD exhibited lower VFT-related average FC strength values in the DLPFC(R) ~ mPFC(R), DLPFC(R) ~ TL(L), and DLPFC(L) ~ TL(L) pathways; however, they exhibited a higher average FC strength value in the DLPFC(L) ~ TL(R) pathway. This finding is notable because it differs from previous findings in adults with MDD, who typically exhibited increased cognitive task-related average FC strength values in the DLPFC(R) ~ TL(L) and DLPFC(R) ~ mPFC(R) pathways [46, 47]. To our knowledge, this divergence may be attributable to the ongoing maturation of the adolescent brain and the influence of depression-related mechanisms during this critical period [48, 49]. Such developmental factors have been found to produce marked differences between adolescent and adult findings [50, 51].

For adolescents with MDD, our study revealed the presence of negative correlations between depressive/anxious symptoms and VFT-related average FC strength value in the DLPFC(R) ~ mPFC(R) pathway. This finding is notable because, even though cognitive task-related fNIRS studies have revealed hypoactivation in the prefrontal and temporal regions in adolescents with MDD [13], the brain activation metric (ΔHbO) in these regions has not been found to correlate with depressive/anxious symptoms [14–16]. The reason might be that the raw ΔHbO is not a specific indicator of cognitive task-related hemodynamic responses. Although the general linear model (GLM) has been employed to estimate the hemodynamic response related to cognitive tasks, assumptions have to be made on temporal parameters of the hemodynamic response function (HRF) [52]. Given the considerable inter-individual differences regarding the HRF’s latency and dispersion, the rigid GLM assumptions can lead to inaccuracies in characterizing neural activity across individuals [53]. In comparison, FC analysis provides a more robust and specific metric for linking neural activity to clinical symptoms, as it is derived from the temporal synchronization between brain regions and is independent of the HRF parameters. Consequently, the correlations we identified between depressive/anxious symptoms and average FC strength in the DLPFC(R) ~ mPFC(R) pathway may better reflect underlying pathophysiological mechanisms of MDD and its progression during adolescence.

Previous research has established the utility of PCA-derived PCs for neurobiological interpretability. For example, Brandon et al. employed the 1st and 2nd PCs derived from the effect profiles of psilocybin and lisuride to characterize correlations between dopamine neuronal activity and behavioral symptoms [22]. Additionally, Kwak et al. conducted a PCA with varimax rotation on cortical thickness and identified that the 1st, 2nd, and 5th PCs were significant predictors of delusion symptoms [54]. Jabes et al. conducted a PCA on resting-state electroencephalographic data of young and older adults, and found that the 2nd and 4th PCs accounted partially for working memory performance [55]. Inspired by these findings, we employed PCA to extract VFT-related dynamic FC features in adolescents with MDD, and the calculated CCRs revealed that the first 6 PCs accounted for approximately 92% of the total variance across participants, demonstrating the suitability of PCA for dynamic FC data. Our experimental results revealed between-group differences in multiple PCs, and identified that the 3rd PC of dynamic FC strength values in the DLPFC(L) ~ TL(L) pathway and the 5th PC of dynamic FC strength values in the mPFC(R) ~ TL(R) pathway were significant predictors of anhedonic symptoms. These findings may provide new insights into the brain mechanisms underlying anhedonia in adolescents with MDD, and demonstrate the feasibility of using PCA-derived PCs of dynamic brain functional data for improving the neurobiological interpretability of psychiatric disorders. Clinically, anhedonia heralds a chronic course of MDD [56]. We therefore expect that the two identified neural predictors of anhedonic symptoms serve as valuable biomarkers for early screening of adolescents who are at a higher risk of developing a chronic course of MDD.

To date, our study has included 83 adolescents with MDD and 78 HCs. The RF model using VFT-related FC features achieved a high accuracy of 86.32% (95% CI: 83.75%-89.38%) for distinguishing adolescents with MDD from HCs. This result indicated that the FC features exhibited by the two groups reflect their specific brain activity responses to VFT or cognitive tasks. This specificity likely stems from adolescents with MDD experiencing functional deficits in key brain regions involved in cognitive tasks, resulting in abnormal FC patterns [57]. In addition to this specificity, the incorporation of static and dynamic FC features into the RF model is essential, as illustrated by the top 12 feature importance ranking in Fig. 6. To further demonstrate the benefits of the incorporation of static and dynamic features into the RF model, we have evaluated the model performance using either static or dynamic FC features separately. The results revealed that the RF model relying on static FC features achieved a classification accuracy of 80.20% (95% CI: 75.00%-83.40%), an F1-score of 0.81 (95% CI: 0.79–0.83), and an AUC of 0.86 (95% CI: 0.83–0.88) on the testing sets across the 10-fold cross-validation process, with an optimal hyper-parameter configuration of 130 decision trees, a minimum leaf size of 1, and a bootstrap fraction of 1. In comparison, the RF model relying on dynamic FC features achieved a classification accuracy of 77.51% (95% CI: 73.34%-82.48%), an F1-score of 0.80 (95% CI: 0.74–0.86), and an AUC of 0.83 (95% CI: 0.79–0.86) on the testing sets across the 10-fold cross-validation process, with an optimal hyper-parameter configuration of 100 decision trees, a minimum leaf size of 1, and a bootstrap fraction of 0.8. As expected, these calculated metrics are consistently lower than those achieved when simultaneously using static and dynamic FC features as the model input.

The present study has three notable limitations. First, the limited sample size (adolescents with MDD = 83, HCs = 78) poses a significant limitation, potentially affecting the robustness of our findings. We will expand the sample size in future studies to enhance statistical power, and furthermore, will conduct independent external validation to assess the RF model’s generalizability. Second, the cross-sectional design inherently limits the ability to infer causality between the identified FC biomarkers and clinical symptoms in adolescents with MDD. Therefore, a future longitudinal validation is essential to confirm the reproducibility and scalability of our findings. Third, all 83 adolescents with MDD were treated with antidepressants before participating in the study. The effects of antidepressants on the FC baseline levels may have introduced bias in the observed experimental results. To mitigate these potential effects, future research will incorporate drug-naive adolescents with MDD to provide more powerful findings.

## Conclusions

This study investigated the abnormalities in VFT-related static and dynamic FC features exhibited by adolescents with MDD, and examined the relationships between these FC features and clinical symptom scores. Our findings demonstrated that the average FC strength value in the DLPFC(R) ~ mPFC(R) pathway was a significant predictor of depressive and anxious symptoms; the 3rd PC of dynamic FC strength values in the DLPFC(L) ~ TL(L) pathway and the 5th PC of dynamic FC strength values in the mPFC(R) ~ TL(R) pathway were significant predictors of anhedonic symptoms. The RF model achieved a high accuracy of 86.32% (95% CI: 83.75%-89.38%) for distinguishing adolescents with MDD from HCs. These results suggest the feasibility of VFT-related static and dynamic FC features as biomarkers for characterizing clinical symptoms in adolescents with MDD. In addition, the RF model could be further developed into an objective tool for diagnostic screening of MDD in adolescents.

## Supplementary Information

Below is the link to the electronic supplementary material.

Supplementary Material 1

## Data Availability

The datasets used and/or analyzed during the current study are available from the corresponding author on reasonable request.

## Abbreviations

MDD: Major depressive disorder
HCs: Healthy controls
FC: Functional connectivity
fNIRS: Functional near-infrared spectroscopy
VFT: Verbal fluency test
PCA: Principal component analysis
PCs: Principal components
DASS-21: 21-item Depression Anxiety Stress Scale
DASS-D: Depression items in DASS-21
DASS-A: Anxiety items in the DASS-21
DASS-S: Stress items in the DASS-21
SHAPS: Snaith-Hamilton Pleasure Scale
ΔHbO: Oxyhemoglobin concentration change
DLPFC: Dorsolateral prefrontal cortex
TL: Temporal lobe
mPFC: Medial prefrontal cortex
R: Right
L: Left
SNR: Signal-to-noise ratio
ROI: Region of interest
FDR: False discovery rate
RF: Random forest
AUC: The area under the curve of the receiver operating characteristic (AUC)
CI: Confidence interval
