# Supplementary material for: fNIRS-Based characterization of adolescent depression using dynamic functional connectivity biomarkers in a verbal fluency task
Source: BMC Psychiatry. 2026 Jan 17;26:150. doi: 10.1186/s12888-026-07799-3 (PMC12895585; doi:10.1186/s12888-026-07799-3)
Supplement: Supplementary file 1 — Supplementary Material 1 [file 12888_2026_7799_MOESM1_ESM.docx]

*Supplemental materials*

**Table 1** Between-group comparisons of average FC strength values.

| Brain pathway | Adolescents with MDD (*n* = 83) | HCs  (*n* = 78) | *t* | FDR-corrected *q* |
| --- | --- | --- | --- | --- |
| DLPFC(R)~TL(R) | 0.18 ± 0.08 | 0.27 ± 0.11 | -1.882 | 0.132 |
| DLPFC(R)~mPFC(R) | 0.30 ± 0.15 | 0.52 ± 0.13 | -3.765 | **0.006** |
| DLPFC(R)~DLPFC(L) | 0.27 ± 0.09 | 0.21 ± 0.09 | 1.402 | 0.235 |
| DLPFC(R)~TL(L) | 0.32 ± 0.12 | 0.50 ± 0.19 | -2.805 | **0.034** |
| DLPFC(R)~mPFC(L) | 0.18 ± 0.07 | 0.28± 0.09 | -2.082 | 0.137 |
| mPFC(R)~TL(R) | 0.20 ± 0.13 | 0.28 ± 0.11 | -1.626 | 0.194 |
| DLPFC(L)~TL(R) | 0.60 ± 0.15 | 0.39 ± 0.15 | 3.467 | **0.008** |
| TL(R)~TL(L) | 0.22 ± 0.09 | 0.26 ± 0.09 | -0.832 | 0.462 |
| mPFC(L)~TL(R) | 0.17 ± 0.16 | 0.26 ± 0.17 | -1.894 | 0.147 |
| DLPFC(L)~mPFC(R) | 0.25 ± 0.09 | 0.34 ± 0.12 | -1.491 | 0.219 |
| mPFC(R)~TL(L) | 0.42 ± 0.17 | 0.50 ± 0.14 | -1.064 | 0.373 |
| mPFC(R)~mPFC(L) | 0.20 ± 0.09 | 0.28 ± 0.10 | -1.936 | 0.157 |
| DLPFC(L)~TL(L) | 0.32 ± 0.07 | 0.67 ± 0.26 | -4.959 | **0.004** |
| DLPFC(L)~mPFC(L) | 0.20 ± 0.07 | 0.31 ± 0.14 | -2.151 | 0.117 |
| mPFC(L)~TL(L) | 0.27 ± 0.09 | 0.27 ± 0.12 | -0.035 | 0.979 |

Data presented as mean ± standard deviation. Significant results (FDR-corrected *q <* 0.05) are highlighted in bold.

Abbreviations: FC, functional connectivity; FDR, false discovery rate; MDD, major depressive disorder; HCs, healthy controls; DLPFC, dorsolateral prefrontal cortex; mPFC, medial prefrontal cortex; TL, temporal lobe; R, right; L, left.

**Table 2** Between-group comparisons of the 1st PCs of dynamic FC strength values.

| Brain pathway | Adolescents with MDD (*n* = 83) | HCs  (*n* = 78) | *t* | FDR- corrected *q* |
| --- | --- | --- | --- | --- |
| DLPFC(R)~TL(R) | 2.16 ± 0.86 | 4.15 ± 1.62 | -3.683 | **0.009** |
| DLPFC(R)~mPFC(R) | 3.38 ± 1.05 | 3.69 ± 1.24 | -0.651 | 0.750 |
| DLPFC(R)~DLPFC(L) | 2.40 ± 0.85 | 5.04 ± 2.17 | -3.853 | **0.006** |
| DLPFC(R)~TL(L) | 3.97 ± 1.21 | 2.88 ± 0.93 | 2.435 | 0.088 |
| DLPFC(R)~mPFC(L) | 3.92 ± 1.37 | 2.44 ± 1.20 | 2.881 | **0.048** |
| mPFC(R)~TL(R) | 3.69 ± 1.26 | 2.12 ± 0.77 | 2.942 | **0.044** |
| DLPFC(L)~TL(R) | 5.09± 1.90 | 2.50 ± 1.07 | 4.031 | **0.004** |
| TL(R)~TL(L) | 3.26 ± 0.96 | 3.92 ± 1.13 | -1.527 | 0.342 |
| mPFC(L)~TL(R) | 3.64 ± 1.31 | 2.49 ± 0.90 | 2.453 | 0.089 |
| DLPFC(L)~mPFC(R) | 3.60 ± 1.47 | 3.79 ± 1.11 | -0.350 | 0.840 |
| mPFC(R)~TL(L) | 3.21 ± 0.85 | 3.59 ± 1.20 | -0.877 | 0.597 |
| mPFC(R)~mPFC(L) | 2.38 ± 0.86 | 3.49 ± 1.51 | -2.299 | 0.101 |
| DLPFC(L)~TL(L) | 3.67 ± 1.06 | 3.42 ± 0.91 | 0.599 | 0.775 |
| DLPFC(L)~mPFC(L) | 3.05 ± 1.23 | 2.50 ± 0.82 | 1.263 | 0.435 |
| mPFC(L)~TL(L) | 2.22 ± 0.69 | 3.41 ± 1.03 | -2.400 | 0.086 |

Data presented as mean ± standard deviation. Significant results (FDR-corrected *q <* 0.05) are highlighted in bold.

Abbreviations: PCs, principal components; FC, functional connectivity; FDR, false discovery rate; MDD, major depressive disorder; HCs, healthy controls; DLPFC, dorsolateral prefrontal cortex; mPFC, medial prefrontal cortex; TL, temporal lobe; R, right; L, left.

**Table 3** Between-group comparisons of the 2nd PCs of dynamic FC strength values.

| Brain pathway | Adolescents with MDD (*n* = 83) | HCs  (*n* = 78) | *t* | FDR-corrected *q* |
| --- | --- | --- | --- | --- |
| DLPFC(R)~TL(R) | 3.68 ± 1.20 | 4.47 ± 1.80 | -1.243 | 0.439 |
| DLPFC(R)~mPFC(R) | 3.48 ± 1.76 | 4.49 ± 1.56 | -1.409 | 0.391 |
| DLPFC(R)~DLPFC(L) | 3.67 ± 1.47 | 3.07 ± 1.45 | 1.092 | 0.514 |
| DLPFC(R)~TL(L) | 3.86 ± 1.49 | 4.54 ± 1.77 | -1.069 | 0.493 |
| DLPFC(R)~mPFC(L) | 3.39 ± 1.36 | 3.19 ± 1.33 | 0.357 | 0.845 |
| mPFC(R)~TL(R) | 4.27 ± 1.40 | 2.96 ± 1.40 | 2.176 | 0.119 |
| DLPFC(L)~TL(R) | 3.29 ± 1.01 | 3.22 ± 1.53 | 0.177 | 0.922 |
| TL(R)~TL(L) | 3.56 ± 1.72 | 2.84 ± 1.12 | 1.160 | 0.482 |
| mPFC(L)~TL(R) | 4.04 ± 1.37 | 4.12 ± 1.46 | -0.150 | 0.933 |
| DLPFC(L)~mPFC(R) | 2.85 ± 0.98 | 3.67 ± 1.73 | -1.433 | 0.384 |
| mPFC(R)~TL(L) | 3.36 ± 1.05 | 3.63 ± 1.42 | -0.511 | 0.775 |
| mPFC(R)~mPFC(L) | 3.00 ± 0.85 | 4.06 ± 1.26 | -2.381 | 0.091 |
| DLPFC(L)~TL(L) | 4.16 ± 1.81 | 4.42 ± 1.40 | -0.408 | 0.822 |
| DLPFC(L)~mPFC(L) | 3.32 ± 1.29 | 4.19 ± 1.64 | -1.575 | 0.353 |
| mPFC(L)~TL(L) | 4.09 ± 1.48 | 4.24 ± 1.48 | -0.234 | 0.895 |

Data presented as mean ± standard deviation. Significant results (FDR-corrected *q <* 0.05) are highlighted in bold.

Abbreviations: PCs, principal components; FC, functional connectivity; FDR, false discovery rate; MDD, major depressive disorder; HCs, healthy controls; DLPFC, dorsolateral prefrontal cortex; mPFC, medial prefrontal cortex; TL, temporal lobe; R, right; L, left.

**Table 4** Between-group comparisons of the 3rd PCs of dynamic FC strength values.

| Brain pathway | Adolescents with MDD (*n* = 83) | HCs  (*n* = 78) | *t* | FDR-corrected *q* |
| --- | --- | --- | --- | --- |
| DLPFC(R)~TL(R) | 3.23 ± 1.22 | 5.18 ± 1.85 | -3.257 | **0.023** |
| DLPFC(R)~mPFC(R) | 3.22 ± 1.25 | 3.29 ± 1.64 | -0.123 | 0.934 |
| DLPFC(R)~DLPFC(L) | 3.89 ± 1.13 | 3.09 ± 1.31 | 1.552 | 0.357 |
| DLPFC(R)~TL(L) | 3.79 ± 1.55 | 3.45 ± 1.88 | 0.501 | 0.763 |
| DLPFC(R)~mPFC(L) | 3.34 ± 0.96 | 4.00 ± 1.65 | -1.278 | 0.444 |
| mPFC(R)~TL(R) | 2.39 ± 1.32 | 3.24 ± 1.27 | -1.757 | 0.255 |
| DLPFC(L)~TL(R) | 3.27 ± 1.08 | 3.15 ± 1.18 | 0.251 | 0.892 |
| TL(R)~TL(L) | 2.58 ± 0.76 | 3.88 ± 1.86 | -2.371 | 0.089 |
| mPFC(L)~TL(R) | 4.51 ± 1.86 | 3.84 ± 1.55 | 0.965 | 0.555 |
| DLPFC(L)~mPFC(R) | 4.28 ± 1.48 | 3.09 ± 1.06 | 2.146 | 0.123 |
| mPFC(R)~TL(L) | 3.03 ± 1.43 | 2.75 ± 1.13 | 0.517 | 0.781 |
| mPFC(R)~mPFC(L) | 3.83 ± 1.40 | 4.27 ± 1.71 | -0.691 | 0.716 |
| DLPFC(L)~TL(L) | 2.42 ± 1.17 | 4.54 ± 1.66 | -4.075 | **0.005** |
| DLPFC(L)~mPFC(L) | 3.91 ± 1.55 | 3.89 ± 1.41 | 0.049 | 0.961 |
| mPFC(L)~TL(L) | 3.82 ± 1.61 | 3.70 ± 1.28 | 0.206 | 0.908 |

Data presented as mean ± standard deviation. Significant results (FDR-corrected *q <* 0.05) are highlighted in bold.

Abbreviations: PCs, principal components; FC, functional connectivity; FDR, false discovery rate; MDD, major depressive disorder; HCs, healthy controls; DLPFC, dorsolateral prefrontal cortex; mPFC, medial prefrontal cortex; TL, temporal lobe; R, right; L, left.

**Table 5** Between-group comparisons of the 4th PCs of dynamic FC strength values.

| Brain pathway | Adolescents with MDD (*n* = 83) | HCs  (*n* = 78) | *t* | FDR-corrected *q* |
| --- | --- | --- | --- | --- |
| DLPFC(R)~TL(R) | 2.39 ± 1.19 | 3.50 ± 0.88 | -2.651 | 0.060 |
| DLPFC(R)~mPFC(R) | 2.74 ± 1.24 | 5.60 ± 1.82 | -4.622 | **0.002** |
| DLPFC(R)~DLPFC(L) | 2.97 ± 0.78 | 3.65 ± 1.49 | -1.552 | 0.346 |
| DLPFC(R)~TL(L) | 2.33 ± 1.09 | 2.69 ± 1.17 | -0.820 | 0.624 |
| DLPFC(R)~mPFC(L) | 3.31 ± 1.41 | 3.84 ± 1.04 | -1.046 | 0.501 |
| mPFC(R)~TL(R) | 3.36 ± 1.06 | 4.91 ± 1.94 | -2.901 | **0.045** |
| DLPFC(L)~TL(R) | 3.49 ± 1.51 | 3.44 ± 1.74 | 0.079 | 0.948 |
| TL(R)~TL(L) | 3.45 ± 1.30 | 4.32 ± 2.09 | -1.314 | 0.438 |
| mPFC(L)~TL(R) | 3.15 ± 1.39 | 3.97 ± 1.56 | -1.453 | 0.381 |
| DLPFC(L)~mPFC(R) | 4.76 ± 1.46 | 2.77 ± 1.59 | 3.139 | **0.028** |
| mPFC(R)~TL(L) | 5.51 ± 1.99 | 4.83 ± 2.03 | 0.864 | 0.598 |
| mPFC(R)~mPFC(L) | 3.54 ± 1.33 | 3.83 ± 1.46 | -0.549 | 0.786 |
| DLPFC(L)~TL(L) | 4.34 ± 1.73 | 3.58 ± 1.81 | 1.072 | 0.493 |
| DLPFC(L)~mPFC(L) | 2.87 ± 1.03 | 2.66 ± 1.25 | 0.457 | 0.790 |
| mPFC(L)~TL(L) | 2.77 ± 0.96 | 3.47 ± 1.57 | -1.537 | 0.346 |

Data presented as mean ± standard deviation. Significant results (FDR-corrected *q <* 0.05) are highlighted in bold.

Abbreviations: PCs, principal components; FC, functional connectivity; FDR, false discovery rate; MDD, major depressive disorder; HCs, healthy controls; DLPFC, dorsolateral prefrontal cortex; mPFC, medial prefrontal cortex; TL, temporal lobe; R, right; L, left.

**Table 6** Between-group comparisons of the 5th PCs of dynamic FC strength values.

| Brain pathway | Adolescents with MDD (*n* = 83) | HCs  (*n* = 78) | *t* | FDR-corrected *q* |
| --- | --- | --- | --- | --- |
| DLPFC(R)~TL(R) | 4.81 ± 1.37 | 4.24 ± 1.54 | 0.938 | 0.567 |
| DLPFC(R)~mPFC(R) | 2.87 ± 0.71 | 3.01 ± 1.45 | -0.346 | 0.832 |
| DLPFC(R)~DLPFC(L) | 3.63 ± 1.36 | 2.38 ± 1.48 | 2.423 | 0.086 |
| DLPFC(R)~TL(L) | 3.74 ± 1.81 | 3.03 ± 1.30 | 1.091 | 0.495 |
| DLPFC(R)~mPFC(L) | 3.02 ± 1.22 | 4.10 ± 1.61 | -1.992 | 0.161 |
| mPFC(R)~TL(R) | 2.02 ± 1.19 | 3.62 ± 0.98 | -2.934 | **0.044** |
| DLPFC(L)~TL(R) | 2.07 ± 1.11 | 3.81 ± 1.35 | -3.013 | **0.032** |
| TL(R)~TL(L) | 3.26 ± 1.40 | 3.11 ± 0.93 | -0.296 | 0.865 |
| mPFC(L)~TL(R) | 6.39 ± 2.42 | 3.92 ± 1.06 | 3.337 | **0.021** |
| DLPFC(L)~mPFC(R) | 3.63 ± 1.40 | 3.07 ± 1.22 | 1.091 | 0.505 |
| mPFC(R)~TL(L) | 4.05 ± 1.39 | 4.09 ± 1.38 | -0.084 | 0.955 |
| mPFC(R)~mPFC(L) | 3.74 ± 1.22 | 2.21 ± 1.77 | 2.942 | **0.048** |
| DLPFC(L)~TL(L) | 3.07 ± 1.29 | 3.40 ± 1.79 | -0.543 | 0.781 |
| DLPFC(L)~mPFC(L) | 3.46 ± 0.96 | 3.19 ± 1.53 | 0.509 | 0.767 |
| mPFC(L)~TL(L) | 2.24 ± 1.08 | 3.54 ± 1.56 | 2.533 | 0.077 |

Data presented as mean ± standard deviation. Significant results (FDR-corrected *q <* 0.05) are highlighted in bold.

Abbreviations: PCs, principal components; FC, functional connectivity; FDR, false discovery rate; MDD, major depressive disorder; HCs, healthy controls; DLPFC, dorsolateral prefrontal cortex; mPFC, medial prefrontal cortex; TL, temporal lobe; R, right; L, left.

**Table 7** Between-group comparisons of the 6th PCs of dynamic FC strength values.

| Brain pathway | Adolescents with MDD (*n* = 83) | HCs  (*n* = 78) | *t* | FDR-corrected *q* |
| --- | --- | --- | --- | --- |
| DLPFC(R)~TL(R) | 3.72 ± 1.32 | 3.03 ± 1.21 | 1.219 | 0.448 |
| DLPFC(R)~mPFC(R) | 3.35 ± 0.95 | 3.66 ± 1.93 | -0.558 | 0.789 |
| DLPFC(R)~DLPFC(L) | 2.81 ± 1.07 | 3.99 ± 1.61 | -2.109 | 0.128 |
| DLPFC(R)~TL(L) | 4.10 ± 1.34 | 3.43 ± 1.65 | 1.132 | 0.493 |
| DLPFC(R)~mPFC(L) | 4.03 ± 1.52 | 4.39 ± 1.86 | -0.593 | 0.770 |
| mPFC(R)~TL(R) | 4.01 ± 1.86 | 3.45 ± 1.27 | 0.919 | 0.572 |
| DLPFC(L)~TL(R) | 3.95 ± 1.37 | 4.72 ± 1.46 | 1.325 | 0.441 |
| TL(R)~TL(L) | 3.48 ± 1.68 | 3.11 ± 1.50 | 0.535 | 0.776 |
| mPFC(L)~TL(R) | 4.59 ± 2.51 | 3.73 ± 1.48 | 1.311 | 0.441 |
| DLPFC(L)~mPFC(R) | 2.48 ± 1.11 | 3.73 ± 1.71 | 2.209 | 0.114 |
| mPFC(R)~TL(L) | 4.01 ± 1.20 | 3.85 ± 1.20 | 0.359 | 0.854 |
| mPFC(R)~mPFC(L) | 3.98 ± 1.56 | 3.27 ± 1.04 | 1.274 | 0.437 |
| DLPFC(L)~TL(L) | 4.18 ± 1.14 | 4.09 ± 1.95 | 0.133 | 0.937 |
| DLPFC(L)~mPFC(L) | 2.10 ± 1.30 | 4.23 ± 1.71 | -4.272 | **0.004** |
| mPFC(L)~TL(L) | 2.68 ± 0.95 | 3.56 ± 1.05 | 2.262 | 0.105 |

Data presented as mean ± standard deviation. Significant results (FDR-corrected *q <* 0.05) are highlighted in bold.

Abbreviations: PCs, principal components; FC, functional connectivity; FDR, false discovery rate; MDD, major depressive disorder; HCs, healthy controls; DLPFC, dorsolateral prefrontal cortex; mPFC, medial prefrontal cortex; TL, temporal lobe; R, right; L, left.
